# Early Fertilization Abnormalities in the Human: An Exhausted Area of Research?

**Published:** 2017

**Authors:** Bernd Rosenbusch

**Affiliations:** Department of Gynaecology and Obstetrics, University of Ulm, Ulm, Germany

## Introduction

The techniques of assisted reproduction have admittedly unveiled the process of human oocyte fertilization and subsequent embryonic development. In specialized laboratories, male and female gametes are routinely handled and their immediate fusion product, the pronuclear stage, can nowadays be cultured up to the hatching blastocyst under *in vitro* conditions. Comprehensive overviews of the observed variable features of oocytes, zygotes, embryos and blastocysts have been provided elsewhere (1–4).

An important side effect of monitoring early events of reproduction is the detection of various factors that may affect regular fertilization and/or cause developmental abnormalities. Quite recently, time-lapse systems (TLS) have been propagated as an ideal tool of continuous monitoring without disturbing the culture conditions and it appears as an immense advantage over the common assessments of pronuclear formation and cleavage at a given time. However, the focus of morphokinetic parameters obtained by TLS has mostly been on embryo development in order to select the “best” embryo and thus improve implantation rates (5).

In the present comment, some issues that delt with the chromosomal constitution of zygotes and embryos produced *in vitro* were focused, evaluating whether TLS could play a role in the early detection of abnormal events. This subjective commentary was inspired for the most part by the knowledge obtained from cytogenetic analyses of abnormally fertilized oocytes in our laboratory. It amplifies the thoughts presented in a recent brief letter (6) and advocates further basic research using and preferably combining so-called morphokinetic aspects and (cyto) genetics during assisted reproduction. Such an approach appears indispensable if the purpose is to shed light on some still ambiguous mechanisms of fertilization disorders.

### Examples for abnormal pronuclear stages:

In one of our case reports, a tripronuclear oocyte resulting from ICSI was reported that turned out to have a tetraploid chromosome constitution (7). Our conclusion was that diploid sperm must exist and being able to fertilize an oocyte through this event had been considered rather improbable by some investigators. Without appearance of an additional female pronucleus (PN) caused by non-extrusion of the second polar body (PB), this phenomenon would have escaped detection. In other words, a diploid spermatozoon that regularly fertilizes an oocyte should result in a single diploid male PN accompanying the haploid female PN. The corresponding bipronuclear stage would be classified as normal and could develop into a diandric triploid embryo. This assumption has recently been confirmed by Savage et al. (8) who applied genotyping to a partial molar pregnancy after ICSI. In the female, there are also diploid gametes that predominantly appear as “giant” oocytes. Two laboratories (9, 10) have independently shown that these oocytes can be fertilized and therefore cause digynic triploidy in the embryo. The problem is that giant oocytes can develop either two or three pronuclei (PNi) after penetration of a spermatozoon, indicating that the abnormality might be overlooked if only the number of PNi is assessed and oocyte size is not taken into account.

In a review on triploidy-causing mechanisms (11), endoreduplication in one of the two PNi has been listed as a possibility because the evidence for this phenomenon was found in the maternal PN of two abnormally fertilized oocytes (12, 13). It is conceivable that endoreduplication can also occur in the male PN. Independent of its parental origin, however, endoreduplication is another good example for the fact that only the number of pronuclei could be assessed and not their genetic content during the routine laboratory procedure.

The formation of a single PN is a peculiar abnormality that should be mentioned here. It is known that some of these monopronuclear oocytes can develop into diploid embryos (14) and result in the birth of normal healthy babies (15). The probability of obtaining a biparental diploid embryo appears to be higher after conventional *in vitro* fertilization (IVF) than after intracytoplasmic sperm injection (ICSI) but births have almost exclusively been reported after transfer of embryos that developed from monopronuclear IVF oocytes. Currently, there is no general consent on how to proceed with monopronuclear oocytes.

Besides deviations from the regular number of PNi and variations in their genetic constitution, there is a rare morphologic abnormality at the pronuclear stage; an early division of the ooplasm that resembles embryonic cleavage. Such a case of premature cytokinesis in an abnormally fertilized oocyte with three PNi was observed before (16). Division of the ooplasm separated one PN from the other two PNi. This event could restore a biparental diploid cell line provided that the detrimental extra PN is excluded and even produce a normal embryo if the haploid cell does not develop further.

### Suggestions for further research:

1) Among the points that still need clarification is the origin of regularly fertilized but monopronuclear oocytes that result in diploid embryos carrying the genomes of both parents. Besides a delayed appearance of the second PN that has been found in 25% of the cases (17), some other mechanisms are under discussion. First, maternal and paternal chromatin are immediately enclosed in a common pronuclear envelope. This process has been shown to occur in mouse oocytes that were injected with spermatozoa close to the metaphase spindle (18). The resulting diploid PN was characterized by a larger size and a greater number of “nucleolus-like bodies”. Second, two individual PNi are formed comparable to the normal fertilization pattern but then undergo a very early membrane fusion. Third, two individual PNi are initially present but one PN experiences a premature breakdown of its envelope (19). It has been proposed that TLS might be useful to elucidate events during the zygote stage (2). In fact, a continuous observation of oocytes from sperm penetration up to zygote formation should provide information on the delayed appearance of PNi, on pronuclear fusion and premature breakdown of the pronuclear membrane whereas immediate enclosure of both parental genomes in a common envelope will possibly escape detection. Moreover, if the purpose is to evaluate the predictive power of pronuclear size, *i.e*. determining whether diploid single PNi are larger than those with a haploid constitution, exact measurements of the pronuclear diameter followed by genetic analyses will be necessary. In cases of presumed premature pronuclear breakdown, it would be interesting to know whether the chromatin lacking a membrane participates in ensuing mitotic divisions. Therefore, the chromosomal constitution of the resulting embryos should be investigated.

2) Fertilization disorders occurring *in vitro* must be carefully screened to reconcile the observed mechanisms with the variety of existing theoretical models for the formation of genetic abnormalities. Let us look for example at the report of Sunde et al. (20) who examined hydatidiform moles showing one biparental cell population and one androgenetic cell population. To explain some of these mosaic cases, the authors suggested “fertilization of one oocyte by one spermatozoon, followed by duplication of the paternal pronucleus, creating a ‘temporary tripronuclear zygote’ with two identical paternal pronuclei ...”. If this statement has to be interpreted as a change from the originally bipronuclear to the tripronuclear state, it should become visible by continuous monitoring of the oocyte. Indeed, a comparable delayed formation of PNi has been described: following the observation of two PNi at about 18 *hr* after injection of a single spermatozoon into an oocyte, some hours later there were four and then even five PNi (21). Though time-lapse imaging has allowed the detection of this unexpected behaviour, it did not provide information on the origin of the additional three PNi. A simple cytogenetic examination of the abnormal pronuclear stage would have been helpful to decide at least whether the number of whole chromosome sets has increased (the chromosomal constitution should then be in the pentaploid range) or whether an approximately diploid constitution is present. In the latter case, splitting of one or both chromatid sets according to our previously published concept (22) has to be considered.

The mechanisms for the development of mosaics proposed by Sunde et al. (20) would further require an “asymmetric cytokinesis”, leading to the distribution of different PNi to different halves of the oocyte (compare figure 4, page 1029 of the corresponding paper). Therefore, more information on the frequency of early division of the oocyte is needed. In the mouse, it was shown that the gentle compression of oocytes during meiotic maturation can lead to the formation of two cells of similar size within the zona pellucida instead of extrusion of a small first PB (23). The authors considered one of these halves to be an extruded large first polar body. Furthermore, both cells could be fertilized by two independent spermatozoa, resulting in the development of twin embryos that may even amalgamate to form a “chimeric hermaphrodite”. A similar concept has been put forward by Winberg et al. (24) who suggested “dispermic fertilization of a parthenogenetically activated oocyte, that is, fertilization by two spermatozoa of two identical haploid ova”. The “presence of both maternal half-genomes and two paternal PNi in the same ‘tetragametic’ zygote” proposed by Boklage (25) may refer to the same phenomenon though an illustration has not been provided here.

As a final example for hypothetical mechanisms that need clarification, I should like to cite the publication by Wegner et al. (26). Here, it was assumed that one of the paternal PNi of a dispermic tripronuclear zygote may remain unreplicated and become distributed to one of the daughter cells of the two cell embryo. At this point, it would fuse with the nucleus of the blastomere and create a triploid cell line whereas the other blastomere would be diploid. Observation of such an abnormal, long-term existence of a PN and its distribution to an embryonic cell would be another challenging task for TLS but of course, ensuing genetic analyses are required to confirm the suspected development of triploidy.

## Conclusion

The examples mentioned at the beginning clearly show that there is a variety of abnormalities at the pronuclear stage that affect the chromosomal constitution of the embryo. Some of the adverse events can be identified by counting the number of PNi whereas others happen within a PN and escape detection. In the latter cases, TLS will obviously not be helpful. On the other hand, it is generally acknowledged that PNi are dynamic structures so that the reliability of their traditional scoring at a fixed and single point in time has to be questioned. TLS may improve our understanding of these early dynamic processes. The continuous observation of fertilized oocytes and developing embryos may also enable us to address some of the unresolved problems discussed above. In this respect, annotating the appearance of individual PNi (tPN1a; tPN2a; tPN3a *etc*.) and their disappearance or fading (tPN1f; tPN2f *etc*.) as proposed by Ciray et al. (27) will be of utmost importance. Observations during this period will help to reveal abnormalities such as the above-mentioned late appearance of extra PNi (21), the phenomenon of three PNi that turn into two (5), or to assess the frequency of premature cytokinesis.

However, possible obstacles and limitations of TLS must be considered. Existing abnormalities of the oocyte, extrusion of the second PB and formation of PNi could be obscured by cumulus cells in case of routine *in vitro* fertilization. The position of the oocyte in the well, the type of microscopy and image acquisition, the available device or software are variables that may restrict the annotation of a desired parameter (27). Furthermore, it cannot be expected that every laboratory will be equipped with time-lapse systems in the near future and those who use it must be willing to spend time for scientific topics including the willingness to communicate their data. Most important, however, abnormal developmental stages should be examined (cyto) genetically.

Due to these numerous prerequisites, relevant data will emerge only slowly. Nevertheless, a more profound monitoring of early fertilization events in conjunction with appropriate genetic methods may open a new and promising chapter in research.
